# Surface plasmon resonance and microscale thermophoresis approaches for determining the affinity of perforin for calcium ions

**DOI:** 10.3389/fimmu.2023.1181020

**Published:** 2023-07-19

**Authors:** Omar Naneh, Mirijam Kozorog, Franci Merzel, Robert Gilbert, Gregor Anderluh

**Affiliations:** ^1^ Department of Molecular Biology and Nanobiotechnology, National Institute of Chemistry, Ljubljana, Slovenia; ^2^ Theory Department, National Institute of Chemistry, Ljubljana, Slovenia; ^3^ Division of Structural Biology, Wellcome Centre for Human Genetics, University of Oxford, Oxford, United Kingdom

**Keywords:** perforin, pore-forming protein, calcium binding, lipid nanodiscs, surface plasmon resonance, microscale thermophoresis, molecular modelling

## Abstract

Perforin is a pore-forming protein that plays a crucial role in the immune system by clearing virus-infected or tumor cells. It is released from cytotoxic granules of immune cells and forms pores in targeted lipid membranes to deliver apoptosis-inducing granzymes. It is a very cytotoxic protein and is therefore adapted not to act in producing cells. Its activity is regulated by the requirement for calcium ions for optimal activity. However, the exact affinity of perforin for calcium ions has not yet been determined. We conducted a molecular dynamics simulation in the absence or presence of calcium ions that showed that binding of at least three calcium ions is required for stable perforin binding to the lipid membrane. Biophysical studies using surface plasmon resonance and microscale thermophoresis were then performed to estimate the binding affinities of native human and recombinant mouse perforin for calcium ions. Both approaches showed that mouse perforin has a several fold higher affinity for calcium ions than that of human perforin. This was attributed to a particular residue, tryptophan at position 488 in mouse perforin, which is replaced by arginine in human perforin. This represents an additional mechanism to control the activity of human perforin.

## Introduction

The pore-forming protein perforin (PFN) (also termed Perforin-1) is an essential part of the vertebrate immune system. Its cytotoxic activity combines innate and adaptive immune systems in the process of eliminating infected and malignant cells via the delivery of granzymes (1). It is produced by cytotoxic lymphocytes, natural killer cells, and cytotoxic T lymphocytes. In cytotoxic lymphocytes, PFN is stored in the acidic and Ca^2+^-poor environment of cytotoxic granules in complexes with serglycin and granzymes, which are proapoptotic serine proteases (2–4). Once the target cell is recognized, an immunological synapse forms between the effector and target cell, and granules become polarized near the synapse, into which their lethal cargo is released (5). Because of its altered environment, PFN subsequently binds to target membranes in a Ca^2+^- (6, 7) and pH-dependent manner (8, 9). Its initial interaction with the membrane is followed by its oligomerization into ~20-mer ring- or arc-like structures that perforate the membrane and form large pores up to 300 Å in diameter (10–13). This process leads to the loss of integrity of the target membrane, initiating processes that lead to the entry of granzymes into the cell, the subsequent induction of apoptosis, and ultimately cell death. The prominent role of PFN becomes evident when its function is impaired by mutations in the PFN gene, genes involved in the regulation of PFN expression, or genes involved in the processing of the precursor form of PFN (14). Mice that lack functional PFN often develop spontaneous lymphomas and are unable to suppress viral or bacterial infections (15, 16). In humans, mutations can lead to severe immune disorders, such as familial hemophagocytic lymphohistiocytosis, juvenile idiopathic arthritis, or macrophage activation syndrome (14, 17, 18).

The pore-forming ability of PFN is determined by the N-terminal membrane attack complex-PFN/cholesterol-dependent cytolysin domain (MACPF/CDC), which has a characteristic, central, D-shaped, four-stranded β-sheet surrounded by amphipathic α-helices that unwind during a drastic conformational change accompanying pore formation (10, 13). The MACPF/CDC domain is followed by the flexible, shelf-like epidermal growth factor domain, which is connected to the pore-forming amphipathic α-helices by a disulfide bond. The C-terminal C2 domain enables the initial Ca^2+^-dependent interaction between PFN and the membrane (9–11). Ca^2+^ ions are bound to PFN in negatively charged pockets formed by aspartate clusters located on the loops at the bottom of the domain (19). Substitutions of these residues, particularly D429, D435, D483, and D485, by alanine causes partial or complete loss of the cytotoxic activity of PFN (7). The crystal structure of mouse PFN revealed two bound Ca^2+^ ions (10). The structure of Ca^2+^-bound SmC2P129, i.e., a C2 domain with the greatest structural similarity to the C2 domain of PFN to date, revealed additional Ca^2+^-binding sites (20). Finally, the crystal structure of engineered C2-domain of mouse PFN, which had four aromatic residues mutated to alanine in order to increase solubility and stability (20), showed five binding sites for Ca^2+^ (19).

Ca^2+^-dependent membrane binding resembles environmental control of the activity of other proteins containing a C2 domain. C2 domains usually contain several Ca^2+^-binding sites with different affinities, consisting of exposed acidic amino acid residues (21, 22). Sites with high affinities are associated with domain stabilization, whereas sites with low affinities promote membrane binding (23, 24). Similarly, Ca^2+^ has been shown to stabilize PFN (20); however, how Ca^2+^ promotes C2 domain interactions with membranes is not yet fully understood. Hemolytic assays of PFN in the presence of various Ca^2+^ concentrations ([Ca^2+^]) indicated that PFN might also possess low-affinity binding sites that promote its activity (6, 7, 25); however, the exact affinities for Ca^2+^ have never been analyzed for the whole-length protein. PFN has an affinity for membranes, complex multidomain structure, and toxic nature and requires intracellular processing, all of which result in low-production yields, which prevent the use of standard biophysical approaches that demand high protein concentrations (26, 27).

To explain the nature of the PFN-membrane interaction as a function of [Ca^2+^], we used a combination of theoretical and experimental approaches. We optimized biophysical experimental approaches, i.e., surface plasmon resonance (SPR) and microscale thermophoresis (MST), which enabled us to estimate the affinity of PFN for Ca^2+^. Using nanodiscs and MST, we developed a new approach to confirm the micromolar affinity of PFN for Ca^2+^ required for PFN interaction with the membrane. Moreover, by using molecular dynamics (MD) simulations, we show that Ca^2+^ ions stabilize the C2 domain of PFN and affect the orientation of PFN to the hydrophobic surface of the membrane. We suggest that the third Ca^2+^ in the PFN structure is required for proper orientation and interaction with the membrane. Ca^2+^-induced exposure of the hydrophobic amino acids on the C2 domain leads to further interactions with the membrane that firmly anchor the protein to the membrane surface. The approaches presented here provide new methods for evaluating the molecular interactions of challenging protein samples and can be applied to other examples of proteins interacting with lipid membranes.

## Materials and methods

### Molecular dynamics simulations

All MD simulations were performed with CHARMM33 and mPFN (PDB ID: 3NSJ), while reducing the number of Ca^2+^ ions from 3 to 0. The mPFN model with two Ca^2+^ ions (2Ca-mPFN) was extracted from published crystallographic data (PDB ID: 3NSJ). In this model one calcium ion is coordinated with D435 and D483 and the other one with D490. In the model without Ca^2+^ ions (apo-mPFN), ions were directly removed from mPFN. In the model with three Ca^2+^ ions (3Ca-mPFN), ions and calcium-binding region-3 were positioned according to the structure of SmC2P1 (PDB ID: 3W5729). In this model the three calcium ions are placed in the grove at the bottom of the C2 domain and are coordinated with D429, D435, D483, D485 and D491. Additionally, in all structures, we explicitly defined 11 disulfide bonds between adjacent Cys residues and missing residue Pro136. The non-bonded interactions were treated using a set of cut-offs. The distance cut-off in generating the list of pairs was set to 13 Å. At 12 Å, the switching function eliminated all contributions to the overall energy from pairwise interactions. At 10 Å, the smoothing function began to reduce a pair’s contribution. The particle-mesh Ewald method (28) was used to treat long-range electrostatic interactions.

Each mPFN structure was solvated in an explicit water environment modeled by the TIP3P water model (29). Protein was centered in an orthogonal simulation cell with the dimensions 170 Å × 100 Å × 70 Å containing approximately 116,000 atoms in total and simulated with periodic boundary conditions. In addition, sodium and chloride ions were added to achieve electro-neutrality of each system at physiological concentrations. To allow water molecules to move into the protein during equilibration, atomic positions were restrained for 200 ps. The 5 ns equilibration period was followed by a 30 ns production run with a time step of 1 fs as an isothermal-isobaric ensemble with a pressure of 1 bar and temperature of 300 K. The flexibility of the simulated proteins was assessed using average mean-square displacement (MSD) (30).

To assess the initial orientation of 3Ca-mPFN regarding the membrane, short MD simulations were performed with a combination of explicit protein structures and implicit membrane models. We used the generalized Born method with simple switching (GBSW) (31) that can take into account the influence of biological membranes. In this case, the membrane is approximated as a solvent-inaccessible, infinite, planar, low-dielectric slab. Equilibrated mPFN structures from all-atom simulations in the solution were brought into contact with this simplified membrane surface at different orientations of the protein axis relative to membrane normal. Two angles, psi (ψ; inclination angle) and phi (φ; rotation around the body axis), were used to sample 300 initial orientations. Individual orientations were randomly chosen within the core of the apex angle psimax = 50° at the bottom of the C2 domain of PFN. For each pre-set initial position, a short, 0.5 ns MD simulation was run for ensemble relaxation of the side chains. The orientation of 3Ca-PFN with the most favorable solvation energy was chosen as a guide to set up the all-atom protein-membrane model.

For all-atom mPFN-membrane MD simulations, we constructed a quadratic POPC bilayer with a length of 120 Å and 211 POPC lipid molecules per layer using CHARMM-GUI (32) together with CHARMM lipid force field (33). The membrane was first equilibrated with 10 μs MD run before setting it in contact with 3Ca-PFN. Initial contact with the membrane was represented by four random orientations deviating slightly (Δψ < 1°) from the guiding orientation predicted by the GBSW method to improve the conformational sampling of PFN binding. As before, the system was immersed in an explicit, electrostatically neutral, physiological solution modeled by a TIP3P water model and sodium and chloride ions. Thus, we obtained a tetrahedral simulation cell with the dimensions 120 Å × 120 Å × 210 Å with approximately 296,000 atoms. Simulations were performed under the same conditions as MDs in solution with an equilibration period of 5 ns and a production run of 20 ns.

The model of hPFN was obtained using I-TASSER (34) with a confidence score (C-score) of 2.00 and a TM score of 0.99 ± 0.04. To visualize the electrostatic potential on the solvent-accessible protein surface, we used Adaptive Poisson-Boltzmann Solver program (35). The simulated systems were visualized using Visual Molecular Dynamics (36) or PyMOL (37).

### Preparation of native human and recombinant mouse perforin and its mutants

Native human PFN (hPFN) was isolated from cytotoxic granules of natural killer cells-like cell line (YT-INDY) and purified by ion metal affinity chromatography, as previously described (26). Cloning and expression of mouse PFN (mPFN) and its mutants was performed as previously described (27). Single amino acid substitutions, D429A, D483A, and W488R, were introduced into the mPFN gene in the pFastBac1 vector by site-directed mutagenesis with a single oligonucleotide (38). The mutagenic oligonucleotides were 5’-AGTAGCGGTCGTGTAAGCTCCCCACAAGTGTTC-3’, 5’-CCAGCCGTAGTCAGCAGCCCAGACCT-3’, and 5’-GATGCTGACTACGGCAGAGACGATGACCTGCTC-3’, respectively. All constructs were verified by nucleotide sequencing. Expression of the proteins was performed in the baculovirus system using Sf9 cells as hosts in 3 L cultures. Briefly, 24 h after infection with corresponding bacmid at a multiplicity of infection of ~5, the supernatant was harvested by centrifugation. The supernatant was then mixed at a ratio of 3:1 vol/vol with buffer containing 150 mM sodium phosphate, 1.2 M NaCl, 3 mM ethylenediaminetetraacetic acid (EDTA), 7.5 mM imidazole, pH 7.4. EDTA-compatible cOmplete His-tag purification resin (Roche, Switzerland) was added to the mixture (0.5 ml per 1 l of culture). After overnight incubation at 4°C on a shaking table, the resin was collected on the Tricorn Empty High Performance column (GE Healthcare, UK). The resin was washed extensively with 20 mM tris(hydroxymethyl)aminomethane (Tris)-HCl, 400 mM NaCl, 5 mM imidazole, pH 7.4, and then with 20 mM 2-(N-morpholino)ethanesulfonic acid (MES), 200 mM NaCl, 300 mM imidazole, pH 5.5. The bound recombinant product was then eluted with 20 mM Tris-HCl, 400 mM NaCl, 500 mM imidazole, pH 7.4. Fractions were analyzed by 4–12% sodium dodecyl sulfate (SDS)-polyacrylamide gel electrophoresis (PAGE) and pooled. The buffer was exchanged against 20 mM Tris-HCl, pH 7.4, and the fractions were concentrated using Amicon Ultra, 10 kDa MWCO (Millipore, USA) centrifugal filtration devices. Final protein concentrations were measured using the Bradford assay (Bio-Rad, USA) according to the manufacturer’s instructions. To evaluate the activity of the products, hemolytic assays were performed in the presence or absence of Ca^2+^ as described below.

### Lipid nanodisc assembly and labelling

The expression and purification of membrane scaffold protein (MSP) and assembly of nanodiscs were performed as previously described (39). The expression plasmid pET-28a with pMSP1E3D1_D73C with a polyhistidine tag (Addgene 39328) was a gift from Dr. S. Sligar. Briefly, for MSP expression, the plasmid was transformed into *Escherischia coli* BL21(DE3) competent cells. The cells were grown at 37°C until A_600_ reached 2.5. Protein production was initiated with the addition of 1 mM isopropyl-β-D-thiogalactoside. The cells were harvested by centrifugation 3 h after the induction. Protein was isolated using Chelating Sepharose FF (GE Healthcare, UK) and additionally purified using size-exclusion chromatography with Superdex 75 (GE Healthcare, United Kingdom). Fractions containing MSP were pooled and concentrated with Amicon Ultra-15 (Millipore, USA).

For nanodisc assembly, 600 μl of 10 mg/ml chloroform-dissolved 1-palmitoyl-2-oleoyl-*sn*-glycero-3-phosphocholine (POPC) and 1,2-dioleoyl-*sn*-glycero-3-phosphoethanolamine (DOPE) labeled with fluorescent dye Atto-647 (DOPEAtto-647) (40:1 mol/mol) were added to a round-bottom flask. A thin lipid film was formed using rotary evaporator Rotavapor R215 (Buchi, Switzerland) and additionally dried using vacuum concentrator Savant SpeedVac DNA110 (Thermo Scientific, USA) at medium drying rate for 3 h. The lipid film was then hydrated by the addition of 560 μl of cholate buffer (20 mM Tris, 25 mM Na-cholate, 140 mM NaCl, pH 7.4), and dissolved with alternating vortexing and heating above the lipid transition temperature using warm running tap water and with a final 15 min sonication in a water bath. To reconstitute the nanodiscs, 250 μl of purified MSP (7 mg/ml) was added, and the mixture was incubated on a bench shaker at 4°C for 3 h. Subsequently, detergent was removed by overnight dialysis of the mixture at 4°C against 3 l of nanodisc buffer (20 mM Tris, 140 mM NaCl, pH 7.4). The mixture was then subjected to size-exclusion chromatography using a Superdex 200 10/30 column (GE healthcare, UK) and eluted with the same buffer. Peak fractions were analyzed with dynamic light scattering to confirm the presence of ~12 nm particles and the homogeneity of the solution. The concentration of nanodiscs was determined using absorbance at 280 nm.

### Monitoring the interaction of Ca^2+^ with perforin by surface plasmon resonance

The affinity of PFN for Ca^2+^ was assessed using Biacore T100 equipped with Series S sensor chip CM5 (Biacore, GE Healthcare). The system was first primed twice with the running buffer (20 mM 4-(2-hydroxyethyl)-1-piperazineethanesulfonic acid (HEPES), 150 mM NaCl, pH 7.4), and hPFN or mPFN and its mutants were immobilized via amine coupling according to the protocol suggested by the manufacturer. Briefly, the surface of flow cells 1 and 2 was first activated with a 30 min injection of 1-ethyl-3-(3-dimethylaminopropyl)carbodiimide hydrochloride/N-hydroxysuccinimide 1/1 mixture at a flow rate of 10 µl/min. Proteins were diluted in 10 mM acetate buffer with 5 mM EDTA, pH 4.5, just prior to the injection and applied over a second flow cell (test flow cell) in four 10 min injections at a flow rate of 2 µl/min. Finally, both flow cells were deactivated with a 15 min injection of 1 M ethanolamine, pH 8.5, at 10 µl/min. The first flow cell, therefore, does not contain any protein and served as a control flow cell in all cases. The final immobilization level of PFN was approximately 2200 response units. The binding of Ca^2+^ ions was determined at two different pH values (7.4 and 5.5) using 20 mM HEPES and 150 mM NaCl. Ca^2+^ was injected in duplicates at concentrations ranging from 0.38 µM to 100,000 µM for 60 s over both flow cells at a flow rate of 20 µl/min, and the dissociation was monitored for an additional 60 s. At the end of each dilution series, the 24.2 µM, 390.6 µM, and 12,500 µM Ca^2+^ injections were repeated, and 100 mM Ca^2+^ injections were repeated several times throughout the experiment to control the surface stability. The regeneration of the surface was not required. All experiments were performed at 25°C. The binding curves were evaluated by using Biacore T100 evaluation Software (Biacore, GE Healthcare Biosciences). All binding curves that are presented in the results section were doubly referenced, i.e. the difference between the test and the control flow cells is shown and the buffer control injection was subtracted from all traces. The K_D_ values were determined with a model of two independent binding sites, as described (40), using Origin 8.1 (OriginLab Corporation).

### Monitoring the interaction of perforin with nanodiscs in the presence of Ca^2+^ ions by microscale thermophoresis

Ca^2+^- or Mg^2+^-dependent protein interactions with labelled nanodiscs were determined using MST. A series of 16 1:1 dilutions of CaCl_2_ were prepared in nanodisc buffer, producing concentrations from 1.53 µM to 50 mM. To measure the dependence of the PFN-nanodisc interaction on ions, labelled nanodiscs were 500× diluted in nanodisc buffer with 0.25 mg/ml bovine serum albumin (BSA). Directly prior to measurement, PFN samples were diluted in nanodisc buffer to 62 nM and admixed to the fluorescent nanodiscs at a 1:1 vol/vol ratio. For the assay, each ion dilution was mixed with the labelled nanodisc-PFN mixture at a 1:1 vol/vol ratio, resulting in final concentrations of 1.6 nM fluorescently labelled nanodiscs, 16 nM PFN, and 0.736 µM–25 mM ions. Each solution was loaded into Monolith NT.115 hydrophilic capillaries (NanoTemper, Germany). Thermophoresis was measured using a Monolith NT.115 instrument (NanoTemper, Germany) at 25°C. Data were evaluated using the signal from the temperature-jump for mPFN and mutants. For hPFN, the equilibrium dissociation constant K_D_ of the interaction was extracted from the fluorescence change within the series. To confirm the titrant-dependent florescence change, we performed the SDS-test. Samples were centrifuged for 5 min at 16,000 × *g* and then denatured by SDS-buffer (4% SDS, 40 mM dithiothreitol) and heating for 5 min at 95°C. Equal fluorescence for the first and last samples from the series was confirmed. All data were evaluated using NanoTemper software.

### Hemolytic activity of perforin and its inhibition with nanodiscs

PFN activity was measured as a change of turbidity of bovine red blood cells, as previously described, with minor modifications (27). To confirm that mPFN interacts with nanodiscs, protein hemolytic activity was measured in the presence or absence of nanodiscs. mPFN was diluted in erythrocyte buffer (20 mM Tris-HCl, 140 mM NaCl, pH 7.4) containing 1 mM CaCl_2_ and 0.31 μM nanodiscs to yield two-fold serial dilutions of 0.01–0.75 µg/ml mPFN. Immediately, an equal volume of washed bovine red blood cells (A_630_ adjusted to 1) were added to the mixtures, and the decrease in turbidity as a function of time was measured using Synergy MX Multi Mode Reader (BioTek, USA).

When testing the range of [Ca^2+^] that supports hemolytic activity of PFN, 50 ng of mPFN and its mutants or 5 ng of hPFN diluted in erythrocyte buffer were admixed with washed bovine red blood cells (A_630_ adjusted to 0.5). Ca^2+^ dilutions prepared in erythrocyte buffer were added to the mixture at a 1:1 vol/vol ratio to yield a final [Ca^2+^] of 2000, 1000, 500, 200, 150, 100, 50, 25, 10, and 5 μM, and then the turbidity of the suspension was measured.

## Results

### Ca^2+^ ions stabilize mouse perforin

**Figure 1:**
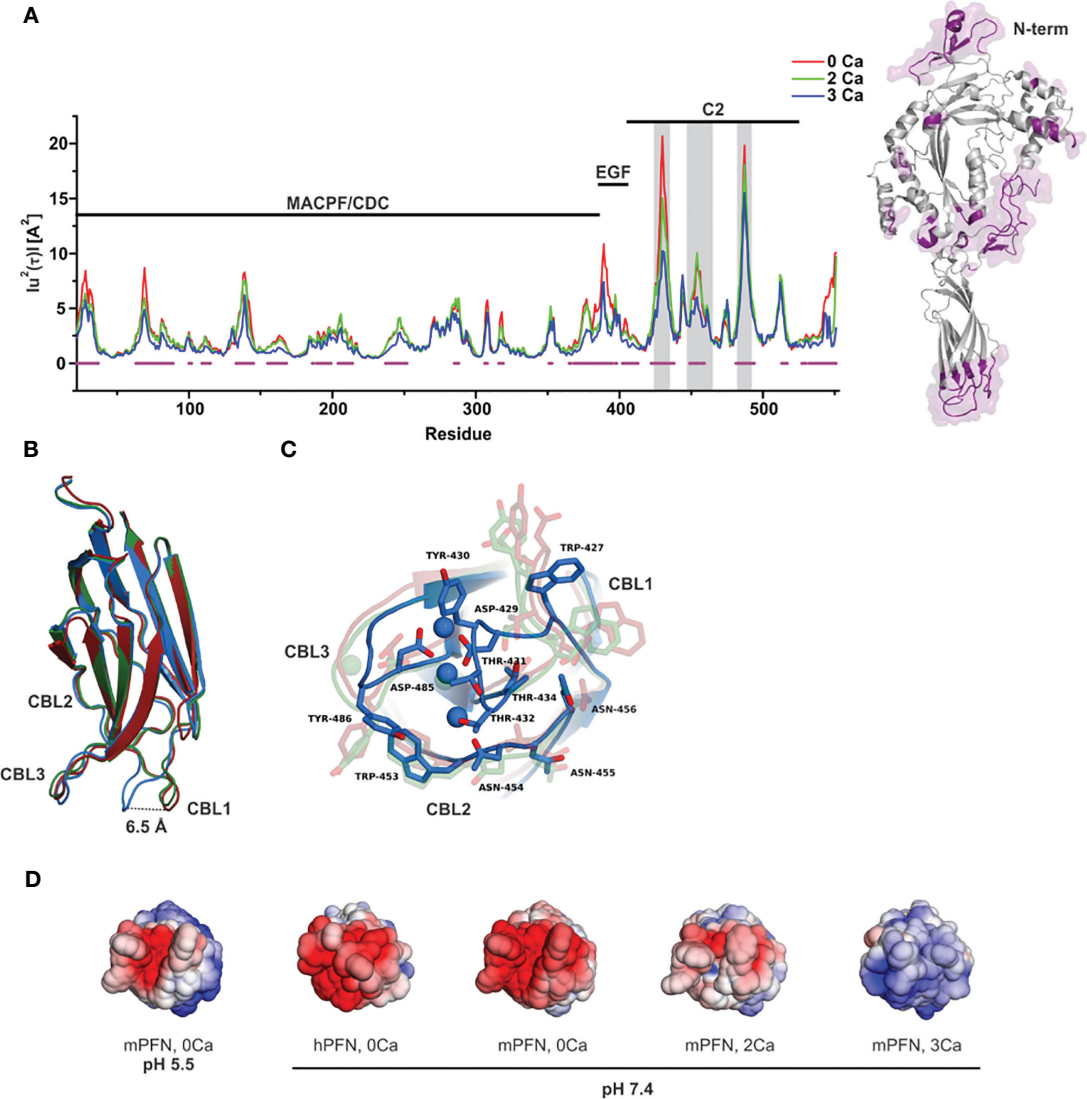
Ca^2+^-induced stabilization of perforin (PFN) and rearrangement of loops that form the ion-binding pocket. **(A)** Mean-square displacement (MSD) of the Cα chain of mPFN from the all-atom 30 ns simulation (red, Apo-mPFN; green, 2Ca-mPFN; blue, 3Ca-mPFN). Calcium-binding loops (CBLs) 1–3 are indicated by gray rectangles. A schematic representation of the structure of mPFN with regions of decreased flexibility in the presence of Ca^2+^ (labelled in magenta) is shown on the right. **(B)** Superposition of C2 domains of all-atom 30 ns simulations. **(C)** The base of the PFN C2 domain with exposed residues with maximal MSD from panel **(B)** Hydrophobic residues, particularly Trp-427, Tyr-430, and Tyr-486, which are believed to be important for interaction with the membrane (20), are less mobile in the presence of Ca^2+^. The labelling in **(B, C)** is as in **(A)**. **(D)** Comparison of ±5 kT/e electrostatic potentials of hPFN and mPFN depending on pH or the number of Ca^2+^ ions bound to the structure. The model of hPFN was obtained using I-TASSER (34). All mPFN structures are the final states of MD simulations.

To quantify the effect of Ca^2+^ ions on the internal dynamics of PFN, we performed whole-atom MD simulations of the structure of mPFN in aqueous solution with different occupancies of the Ca^2+^-binding sites. The average MSD of an atom or a group of atoms has been shown to be a useful fundamental dynamical quantity for characterizing the internal flexibility of modelled proteins (30). Here, we have calculated MSD based on the internal dynamics of PFN in different Ca^2+^-binding states for the backbone CA atoms separately for the entire protein (**Figure 1A**). It should be noted that internal dynamics imply a subtraction of rigid body motion (translation and rotation) of an investigated object from the MD trajectory. Therefore, peaks in **Figure 1A** denote residues of increased flexibility. Furthermore, **Figure 1A** shows an evident decrease of peaks with increased numbers of bound Ca^2+^, indicating a stabilizing role of ions on mPFN dynamics. The effect is mostly localized to the C2 domain. In particular, MSD shows a significant drop for residues 429–435 and 483–488, corresponding to the Ca^2+^-binding loops (CBLs) CBL1 and CBL3, which embrace the Ca^2+^-binding region (**Figures 1B, C**). This drop is accompanied by the closing of the Ca-binding region through a major shift of CBL1 and a minor shift of CBL3 (**Figure 1B**). Surprisingly, also the N-terminal part of the MACPF domain seems to be less flexible, suggesting that bound ions have a long-range stabilizing effect over the entire mPFN structure (**Figure 1A**). In addition, the epidermal growth factor domain seems to be significantly stabilized in the presence of Ca^2+^ ions by interactions with the unstructured C-terminal part of PFN, which follows the C2 domain of PFN (**Figure 1A**).

In C2 domains, highly conserved aspartate residues, together with main-chain oxygen atoms of some other residues, form an electronegative region required for Ca^2+^ binding (41). Similar to others, the Ca^2+^-binding region of the PFN C2 domain is shaped by aspartate residues 429, 435, 483, and 485, which are significant for the hemolytic activity of PFN (7). In agreement with this, we observed high electronegativity on the surface of the C2 domain of modelled mPFN in the absence of Ca^2+^ (**Figure 1D**). We also observed reduced electronegative potential on the mPFN C2 domain at pH 5.5. This implies that the interaction of PFN with Ca^2+^ depends on pH due to the effects on the charge of the Ca^2+^-binding region. hPFN has high (~68%) identity with mPFN. Using the known crystal structure of mPFN (10), we built a model of hPFN that also shows an electronegative surface at the bottom of the C2 domain, implying similar pH-dependent Ca^2+^ affinity. Moreover, the interaction of C2 domains with membranes, particularly zwitterionic or negatively charged membranes, strongly depends on the charge of residues located on CBLs that are altered by the presence of Ca^2+^ (42). Similarly, we observed a Ca^2+^-dependent increase in positive charge on the surface of C2 domains of modelled mPFN structures with different numbers of Ca^2+^ (**Figure 1D**), suggesting an analogous model of PFN interaction with lipid membranes.

### At least three Ca^2+^ ions are required to strengthen the interaction between mouse perforin and the hydrophobic lipid surface

**Figure 2:**
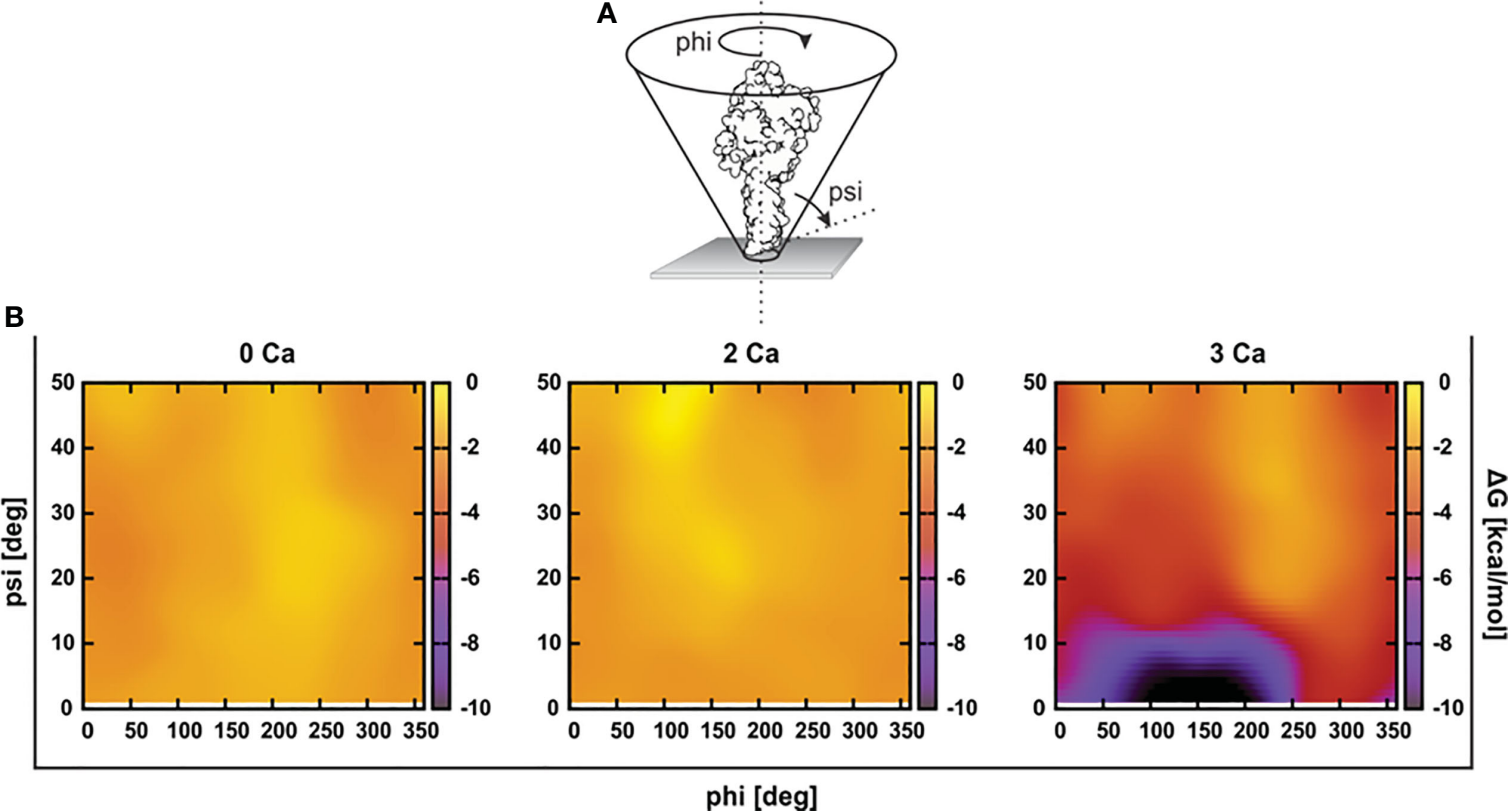
At least three Ca^2+^ are required for favorable interaction of the perforin (PFN) monomer with the hydrophobic plane. **(A)** Structures of Apo-mPFN, 2Ca-PFN, and 3Ca-PFN were brought into the proximity of an implicit dielectric plane at different angles to the normal of the plane. **(B)** Subsequently, short (0.5 ns) simulations were performed, and the interaction of implicit membrane and PFN was analyzed as a function of PFN incline. The interaction is represented as an energy plane in which energy minima denote the energetically most favorable incline of PFN with regard to the membrane.

To assess the initial interaction of PFN with the membrane, we estimated an optimal orientation of PFN relative to the membrane surface normal by simulations in the presence of implicit solvent. We calculated the solvation energies of mPFN as a function of the angles psi (incline to the membrane normal) and phi (rotation around the body axis of mPFN) (**Figure 2**). The results demonstrate a clear preference for the perpendicular orientation of 3Ca-mPFN (three bound Ca^2+^ ions) relative to the dielectric solvent, whereas 2Ca-mPFN (two bound Ca^2+^ ions) and Apo-PFN show only negligible tendency to bind with the membrane with no significant orientational preference. These results imply that at least three Ca^2+^ ions, coordinated as in SmC2P1 (20), are required for mPFN to interact with the membrane in an energetically favorable manner.

### Mouse perforin forms weak but steady interactions with membranes through its β-groove

**Figure 3:**
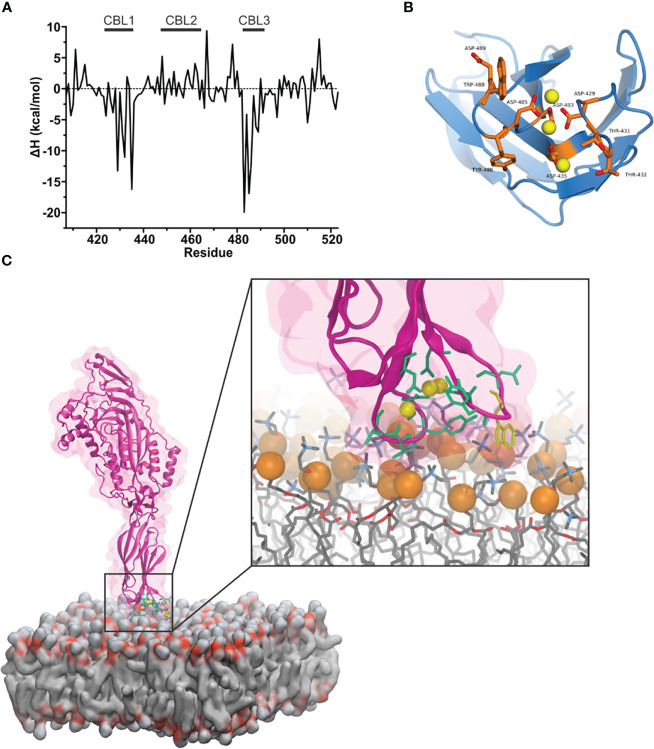
Mouse perforin (mPFN) forms steady interactions with the membrane through calcium-binding loops (CBLs). **(A)** Binding enthalpy of the mPFN C2 domain calculated from simulated 3Ca-mPFN in the presence of a POPC bilayer. **(B)** Exposed residues (orange) on the bottom of the mPFN C2 domain that have minimal binding enthalpy according to the data in **(A)**. **(C)** Simulation snapshot of the interaction between mPFN and the membrane with exposed residues (green and yellow Trp-488), according to the data in **(A)**.

Ca^2+^ facilitates the binding of proteins with C2 domains through two steps: (i) by neutralizing the electronegative cleft and bridging the domain with the membrane through electrostatic interactions and (ii) by exposing the hydrophobic amino acids that serve to bind with lipids of the membrane. Hydrophobic amino acids at the bottom of the mPFN C2 domain (Trp-427, Tyr-430, Tyr-486, and Trp-488) are required for mPFN activity, suggesting their role in anchoring the protein to the membrane prior to pore formation (20). We performed all-atom simulations of 3Ca-mPFN in the presence of POPC membranes. As a guide to construct an all-atom model of 3Ca-mPFN in contact with a POPC membrane, we used the best result regarding the PFN orientation obtained in the explicit-implicit simulation (**Figure 2**). To improve the conformational representation of the mPFN-membrane contact, we simulated the system with four different relative orientations to the membrane. Besides the orientation proposed by the GBSW method, we chose three additional orientations that slightly deviated from the first. None of the four 30 ns long MD runs demonstrated a deep penetration of PFN into the membrane below the phosphate groups of POPC. PFN stayed on the surface by forming relatively strong and stable interactions with the membrane through CBL1 and CBL3, particularly with hydrophobic residues (**Figure 3**). This indicates that such surface interaction could allow PFN to laterally drift on the membrane and exhibit higher degrees of freedom, thus enabling PFN to interact with other monomers during the first steps of pore formation.

Furthermore, we characterized the binding of 3Ca-mPFN to the membrane by calculating the binding enthalpies per residue relative to mPFN in aqueous solution. The binding enthalpy of residue i, ΔH_i_, is given as the sum of the difference in the potential energy of residue i when mPFN is in contact with the membrane versus only immersed in water. Individual potential energies were averaged during the last 5 ns of each MD run. We observed that favorable changes in enthalpy (**Table 1**) predominantly take place in CBL1 and CBL3 (**Figures 3A, B**). The largest drop in binding enthalpy, indicating stronger interactions, occurred through the aspartate residues that interact with Ca^2+^, i.e., Asp-483, Asp-485, Asp-435, Asp-429, and Asp-489 (ordered from highest to lowest), suggesting stable coordination of ions. Regarding binding to the membrane, binding enthalpy particularly decreases with residues Tyr-486, Trp-488, and Trp-427, but not Tyr-430. Additionally, the adjacent residues Thr-431 and Thr-432 have significantly decreased enthalpy. Collectively, these results confirm that hydrophobic residues at the bottom of the PFN C2 domain are involved in the initial interaction of PFN monomers with the membrane.

**Table 1:** Binding enthalpies.

Transition of the state*	ΔH*_i_* (kcal/mol)
Apo-mPFN(s) to 2Ca-mPFN(s)	−158.57
2Ca-mPFN(s) to 3Ca-mPFN(s)	−55.59
3Ca-mPFN(s) to 3Ca-mPFN(m)	−125.88
Apo-mPFN(s) to 3Ca-mPFN(s)	−214.16
Apo-mPFN(s) to 3Ca-mPFN(m)	−340.04

* (s) = simulation in solution, (m) = simulation in the presence of the membrane.

### The affinity of perforin for Ca^2+^ is pH-dependent—a surface plasmon resonance approach

**Figure 4:**
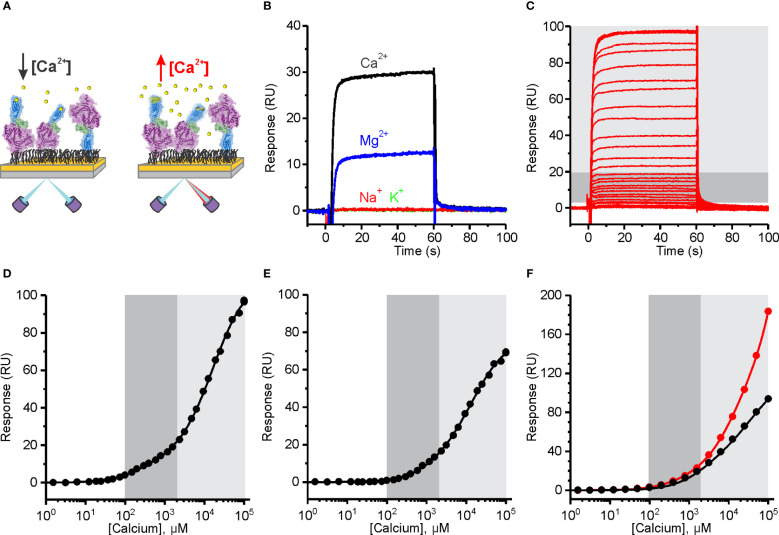
The affinity of Human perforin (hPFN) for Ca^2+^ is in the micromolar range and pH-dependent. **(A)** A schematic representation of the experiment. PFN was directly bound to the dextran-coated CM5 SPR chip in the presence of EDTA and then extensively washed with 20 mM HEPES, 150 mM NaCl, pH 7.4. **(B)** Reponses observed upon injection of 5 mM Ca^2+^ and Mg^2+^, and 20 mM Na^+^ and K^+^. **(C)** Sensorgrams of the interaction between hPFN and Ca^2+^ titrated at 0.38 μM–100 mM (from the bottom to the top). **(D–F)** Steady-state values of the hPFN-Ca^2+^ interaction at pH 7.4 **(D)** and 5.5 **(E)** compared to the BSA-Ca^2+^ interaction (black) or titration over the non-modified flow cell with carboxyl groups (red) at pH 7.4 **(F)**. Models with two independent binding sites were fitted against [Ca^2+^]-dependent steady-state values and are shown as solid lines in each panel. From the models, K_D_ values were estimated as described (40). The high-affinity region (0.1–2 mM; dark gray) and low-affinity region (> 2 mM; light gray) are denoted **(C–F)**.

We developed an SPR assay to evaluate the ion-binding capabilities of PFN. MD simulations indicate that Ca^2+^ ions induce conformational changes and rigidification of the PFN molecule (**Figure 1**). We therefore expected to detect [Ca^2+^]-dependent changes in the SPR signal, which could result from the change in mass upon binding of Ca^2+^ ions and structural changes of the PFN molecule (**Figure 4A**). We immobilized relatively high amounts of hPFN (approximately 2200 response units) to the dextran layer of a CM5 sensor chip in the presence of ion-chelators and then injected a range of [Ca^2+^] over the sensor chip.

**Figure 5:**
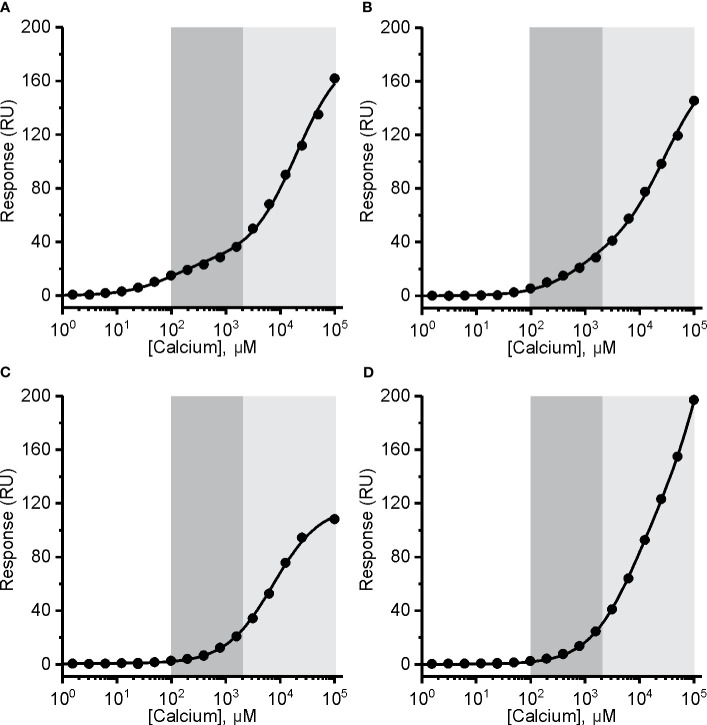
The affinity of mouse perforin (mPFN) for Ca^2+^ is in the micromolar range and pH-dependent. Interaction of Ca^2+^ with mPFN at pH 7.4 **(A)** and pH 5.5 **(B)** and with mutants D429A **(C)** and D483A **(D)** (at pH 7.4). The high-affinity region (0.1–2 mM; dark gray) and low-affinity region (> 2 mM; light gray) are denoted. Steady-state values are shown dependent of [Ca^2+^]. Models with two independent binding sites were fitted against [Ca^2+^]-dependent steady-state response values and are shown as solid lines in each panel.

PFN bears highly specific Ca^2+^-dependent activity (25). In this assay, we observed increases in SPR responses in the presence of 5 mM Ca^2+^, whereas injection of 20 mM Na^+^ or K^+^ did not alter responses (**Figure 4B**), indicating that these monovalent ions do not specifically interact with PFN. We also see increases in the signal upon injection of 5 mM Mg^2+^, however, they were much lower than in the case of Ca^2+^ (**Figure 4B**). Sensorgrams showed fast association and dissociation rates of divalent ions binding. Titration over a range of [Ca^2+^] produced a biphasic dose-dependent response (**Figure 4C**). From the plotted steady-state binding levels against [Ca^2+^], two saturation levels are clearly apparent: at ~1 mM Ca^2+^ (high-affinity region; dark gray area in **Figures 4C–F**) and at > 2 mM (low-affinity region; light gray area in **Figures 4C–F**). We assumed that the high-affinity region describes specific Ca^2+^ binding to the C2 domain, whereas the low-affinity region could correspond to the binding of Ca^2+^ ions to other negatively charged groups on the protein. We, therefore, included additional controls in order to check for this. First, we immobilized the negative control (BSA) at the same levels as PFN and no saturation levels were observed in the low-affinity region, whereas the SPR signal increased upon high [Ca^2+^] injections (black curve on **Figure 4F**). Then we also measured responses when the flow cell was left unreacted, bearing a high density of carboxyl groups on its surface (the control flow cell was treated the same as in the case of PFN, therefore, it was blocked and did not bear any exposed carboxyl groups). In agreement, the responses were quite high in comparison to any other condition at high [Ca^2+^] (see other graphs on **Figures 4**, **5**) and no saturation levels were apparent in the low-affinity region (red curve on **Figure 4F**).

We then treated the PFN titration data by using the previously described model of two independent binding sites (40) and calculated the K_D_ values of interactions. K_D_ values for native hPFN at pH 7.4 were thus 380 ± 130 μM and 20 ± 5 mM for the high- and low-affinity sites, respectively (**Figure 4D** and **Table 2**).

**Table 2:** Equilibrium dissociation constants (K_D_) as determined by surface plasmon resonance and microscale thermophoresis.

Protein	Surface plasmon resonance		Microscale thermophoresis
	High-affinity sites	Low-affinity sites	
hPFN, pH 5.5	1540 ± 390 μM (n=7)	50 mM	n. m.
hPFN, pH 7.4	380 ± 130 μM (n=6)	20 ± 5 mM	123 ± 33 μM (n=3)
mPFN, pH 5.5	643 ± 78 μM (n=3)	25 mM	n. m.
mPFN, pH 7.4	101 ± 13 μM (n=3)	22 ± 4 mM	26 ± 5.8 μM (n=6)
mPFN D429A, pH 7.4	-	8.4 ± 1 mM	n. b.
mPFN D483A, pH 7.4	-	> 50 mM	n. b.
mPFN W488R, pH 7.4	128 ± 22 μM (n=3)	18.5 ± 3.5 mM	160 ± 18.3 μM (n=3)

hPFN, human perforin; mPFN, mouse perforin; n.b., no binding observed; n.m., not measured; -, only binding in low-affinity region is observed. Number of experiments is shown in parentheses.

A specific feature of PFN, which is not common to other known MACPF proteins, is its pH-dependent activity (8, 9). Proteins with C2 domains coordinate Ca^2+^ ions through negatively charged residue side-chains. The charge of these side-chains in PFN is defined by pH (**Figure 1D**), and thus we suspect that decreasing pH could decrease the affinity of PFN for Ca^2+^. Indeed, at pH 5.5, the affinity of high-affinity sites for Ca^2+^ significantly decreased. At pH 5.5, in the case of hPFN, the first saturation level was less apparent (**Figure 4E**), and the K_D_ of high-affinity sites was lower: K_D_ = 1540 ± 390 μM in comparison to pH 7.4 (**Table 2**).

In the case of recombinant mPFN, we observed a higher affinity for Ca^2+^: 101 ± 13 μM and 22 ± 4 mM for high- and low-affinity sites, respectively (**Figure 5A** and **Table 2**). Similar to hPFN, we also observed reduced affinity of mPFN for Ca^2+^ at pH 5.5, with a K_D_ value of 643 ± 78 μM for the high-affinity site (**Figure 5B**). We used two aspartate mutants, D429A (**Figure 5C**) and D483A (**Figure 5D**), to check the binding specificity. Similar to the BSA and empty flow cell titration (**Figure 4F**), we did not observe any saturation levels in the high-affinity region for any of the mutants (**Figures 5C, D**). The mutants additionally confirm that the interactions resolved from the SPR measurements involve Ca^2+^ binding to the β-groove of the PFN C2 domain.

### Binding of perforin to nanodiscs occurs in the presence of Ca^2+^—a microscale thermophoresis approach

**Figure 6:**
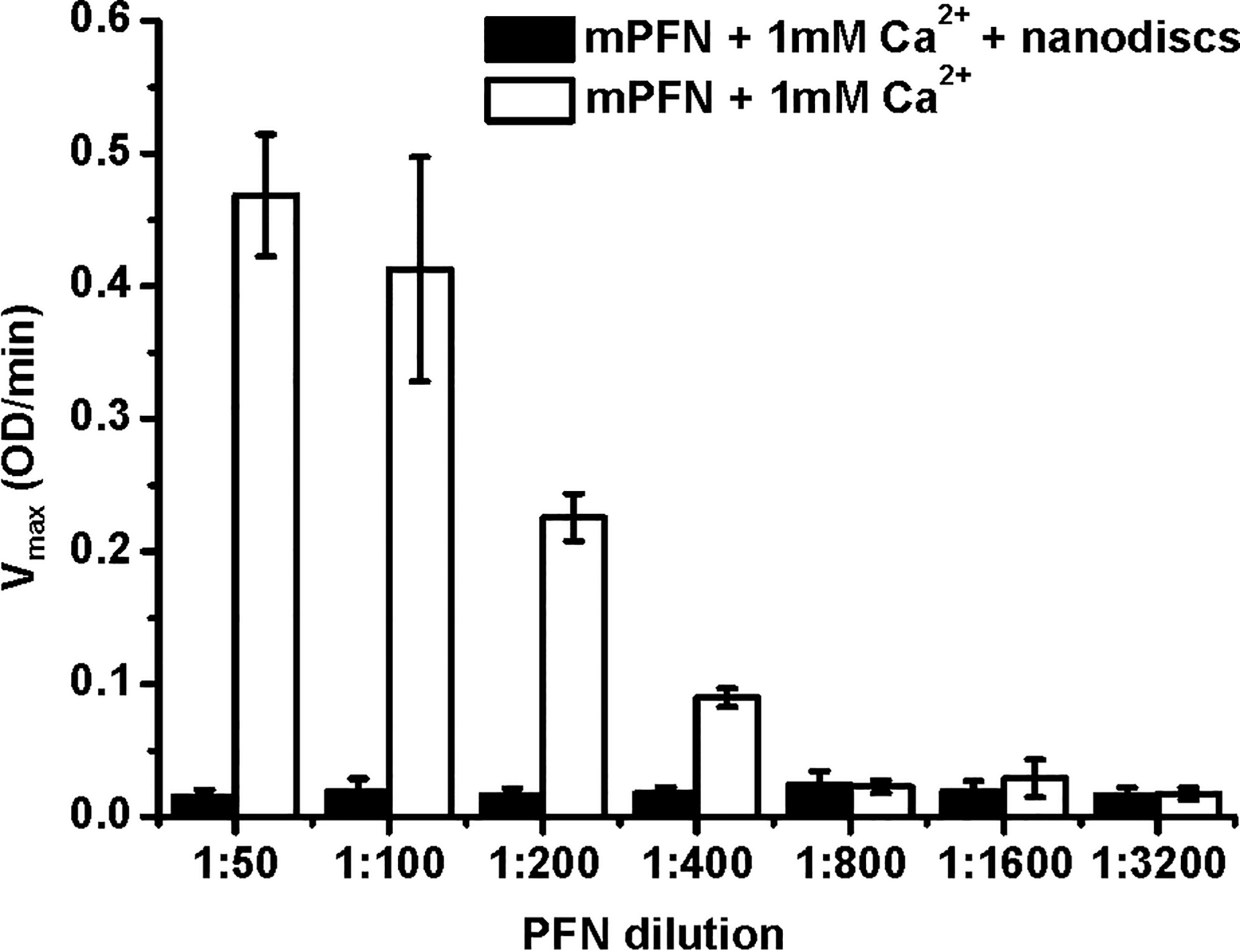
Mouse perforin (mPFN) interacts with nanodiscs in the presence of Ca^2+^. The maximum rate of hemolysis of mPFN in the presence of nanodiscs. The erythrocyte suspension was added to two-fold dilutions of mPFN in buffer with or without nanodiscs and 1 mM Ca^2+^, and the decrease in the rate of change in turbidity was measured. The result is shown as the mean ± standard deviation of three independent measurements.

To develop a lipid membrane model system for an MST approach, we generated nanodiscs with a Stokes hydrodynamic diameter of ~12 nm (43). The membrane surface of nanodiscs cannot provide enough lipid surface to support larger PFN oligomers or a full PFN pore with a lumen diameter of ~15 nm. Therefore, PFN could only attach to the surface of nanodiscs in a monomeric or small-oligomeric form. We used these lipid nanodiscs to further analyze the initial interactions of PFN with the membrane and Ca^2+^ dependence. To confirm mPFN binding to the nanodiscs, we analyzed hemolytic activity of mPFN in the absence and presence of nanodiscs. The pre-incubation of different mPFN dilutions with a saturating concentration of nanodiscs abolished the hemolytic activity of mPFN (**Figure 6**) due to stable binding of mPFN to nanodiscs.

**Figure 7:**
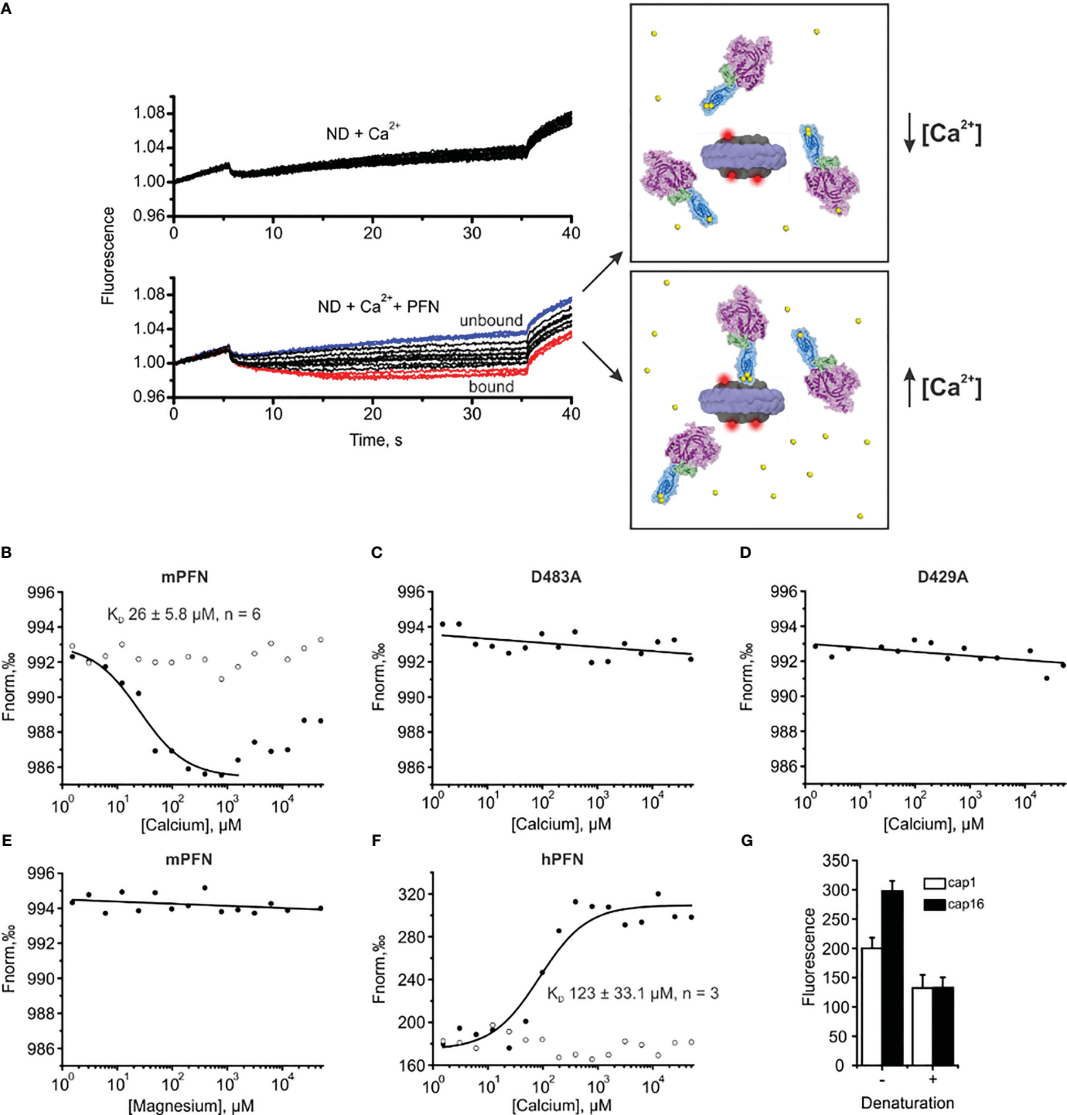
Microscale thermophoresis (MST) assay enables the estimation of the affinity of perforin (PFN) for Ca^2+^ in the presence of lipid nanodiscs (NDs). **(A)** Raw time traces of the experiment in the absence or presence of PFN (left) and schematic interpretation of the MST experiment (right). **(B–F)** Representative binding curves for mPFN **(B)**, mPFN D483A mutant **(C)**, mPFN D429A mutant **(D)**, mPFN in the presence of magnesium ions **(E)**, and hPFN **(F)**. Open and solid circles represent conditions in the absence or presence of Ca^2+^ ions, respectively. **(G)** The fluorescence in capillaries 1 (1.53 µM CaCl_2_) and 16 (50 mM CaCl_2_) before and after denaturation. The samples were denatured by the addition of 4% SDS and 40 mM dithiothreitol and by heating for 5 min at 95°C.

To determine the effective [Ca^2+^] that is required for the interaction of active PFN with lipid membrane, we developed a three-component MST-based assay in which fluorescently labelled nanodiscs were used. Chemical labelling of mPFN, required for MST-based assays, abolished its hemolytic activity (data not shown), and thus we incorporated ~3 DOPE molecules labelled with fluorescent dye Atto-647 into the POPC-MSP nanodiscs. Excess PFN, compared to the nanodiscs, was decreased to a level at which no self-aggregation of bound PFN in the presence of Ca^2+^ occurred during the MST measurement. Therefore, to obtain an effective affinity of PFN for Ca^2+^, we titrated Ca^2+^ into a mixture and measured the fluorescence change before and during MST analysis. We assumed that in the presence of sufficient [Ca^2+^], PFN would bind to the phospholipids of nanodiscs and consequently influence the fluorescence properties or movement of fluorescently labelled particles in the thermophoretic field (**Figure 7A**).

Upon applying a temperature gradient, the fluorescence of the reaction solution changed due to the movement of labelled nanodiscs. The movement was not [Ca^2+^]-dependent when PFN was not present in the solution (**Figure 7A**). Similarly, the presence of PFN did not significantly affect the fluorescent signal at low [Ca^2+^], as PFN did not interact with the nanodiscs (upper right panel and blue time traces in the lower left panel in **Figure 7A**). At higher [Ca^2+^], PFN altered fluorescent signals, most likely by binding to the nanodiscs and thus increasing the size of the complex and hydration shell. Consequently, movement of nanodiscs within the temperature gradient changed (lower right panel and red time traces in the lower left panel in **Figure 7A**).

In agreement with previous observations that phospholipids enhance the Ca^2+^ binding of C2 domains (24, 44, 45), we observed an increased affinity of mPFN for Ca^2+^ in the presence of nanodiscs. Compared to the SPR data, for which affinity for Ca^2+^ was tested in the absence of lipids, the MST results regarding mPFN show a ~six-fold increase of K_D_ to 26 ± 5.8 μM (n=6) (**Figure 7B** and **Table 2**). The mPFN mutants with abolished Ca^2+^ binding, D429A and D483A, did not significantly alter the fluorescence signal in the presence of Ca^2+^ (**Figures 7C, D**). Generally, the binding of proteins with C2 domains is Ca^2+^-specific, and thus we did not observe any significant change of thermophoretic behavior of fluorescent nanodiscs when Mg^2+^ was titrated against a nanodisc-mPFN mixture (**Figure 7E**). Unlike with mPFN, we observed significant [Ca^2+^]-dependent changes in nanodisc fluorescence intensity upon introduction of hPNF into the mixture (**Figure 7F**). To confirm that the altered fluorescence is due to a binding event, we denatured the samples with the lowest and highest [Ca^2+^] and re-measured the fluorescence of the solutions. As expected, after protein denaturation, fluorescence intensity was restored due to the dissociation of denatured hPFN from the fluorescent probe (**Figure 7G**), indicating that the fluorescence change is due to the association of hPFN with nanodiscs. After fitting the fluorescence-signal data, KD = 123 ± 33 μM (n=3) for hPFN (**Figure 7F** and **Table 2**). As with mPFN, this indicates that lipids enhance (by ~2.2-fold) the Ca^2+^-binding properties of hPFN.

### The lower affinity of human perforin for Ca^2+^ in comparison to mouse perforin can be attributed to the residue 488

**Figure 8:**
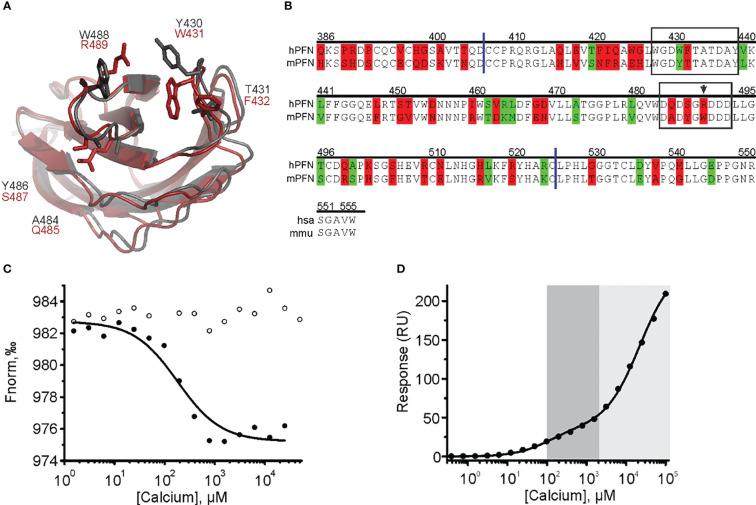
The cation clefts of the human perforin (hPFN) and mouse perforin (mPFN) C2 domain and the contribution of human R489 (mouse W488) to calcium binding. **(A)** C2 domains of mPFN (*grey*) and hPFN (*red*) and the variable amino acids. **(B)** Sequence alignment of hPFN and mPFN at the C-terminal end. Identical residues are not labeled. Residues with and without altered amino acid biophysical properties are highlighted in red and green, respectively. The following is also indicated: CBL1 and CBL3 (*frames*), W488 (*arrow*), and C2 domain boundaries (*blue lines*). Position numbering of amino acid positions is based on mPFN. **(C)** A representative binding curve of mPFN W488R for Ca^2+^ ions from the microscale thermophoresis experiment with and without Ca^2+^ (*solid* and *open circles*, respectively). **(D)** Representative steady-state SPR responses at different [Ca^2+^] of three independent experiments. Interaction affinities are given as K_D_, as the mean of three measurements ± standard deviation. The high-affinity region (0.1–2 mM; *dark gray*) and low-affinity region (> 2 mM; *light gray*) are denoted on the graph.

Both biophysical approaches revealed that hPFN has a lower affinity for Ca^2+^ compared to mPFN (**Figures 4**, **5**, **7**). However, the Ca^2+^ sites of the proteins do not differ in their aspartate residues responsible for coordinating Ca^2+^ (**Figures 8A, B**). Nevertheless, hPFN has a positively charged arginine instead of hydrophobic tryptophan in CBL3 (**Figures 8A, B**). To assess whether this positively charged residue affects the affinity of hPFN for Ca^2+^, we expressed mPFN mutant W488R. The major obstacle in measuring the affinity of W488R was that we did not detect a statistically significant change in nanodisc fluorescence when we used the same protein concentration as in the MST experiments with mPFN or hPFN. The MD simulation revealed that besides other hydrophobic residues at the bottom of the mPFN C2 domain, W488 plays an important role in anchoring the protein to the membrane surface. Therefore, we assumed that the change of a hydrophobic residue to a charged residue decreases the affinity of PFN for uncharged membranes. Based on this assumption, we investigated interactions with nanodiscs at a slightly higher (2.5×) concentration of mutant PFN compared to mPFN. As expected, the mutant possessed markedly lower (~six-fold) affinity compared to mPFN (K_D_= 160 ± 18.3 μM; **Figure 8C** and **Table 2**), confirming that arginine is to a large extent responsible for the lower affinity of hPFN for Ca^2+^. We also estimated the affinity for Ca^2+^ with the SPR approach and observed increased K_D_ of the high-affinity site: K_D_= 128 ± 22 μM (**Figure 8D** and **Table 2**). This indicates that positively charged arginine only slightly affects the Ca^2+^-binding capabilities of hPFN in solution, and that neutral lipids do not affect the mutant’s ion-binding capabilities.

### Hemolytic activities of human and mouse perforin differ in their [Ca^2+^] requirements

**Figure 9:**
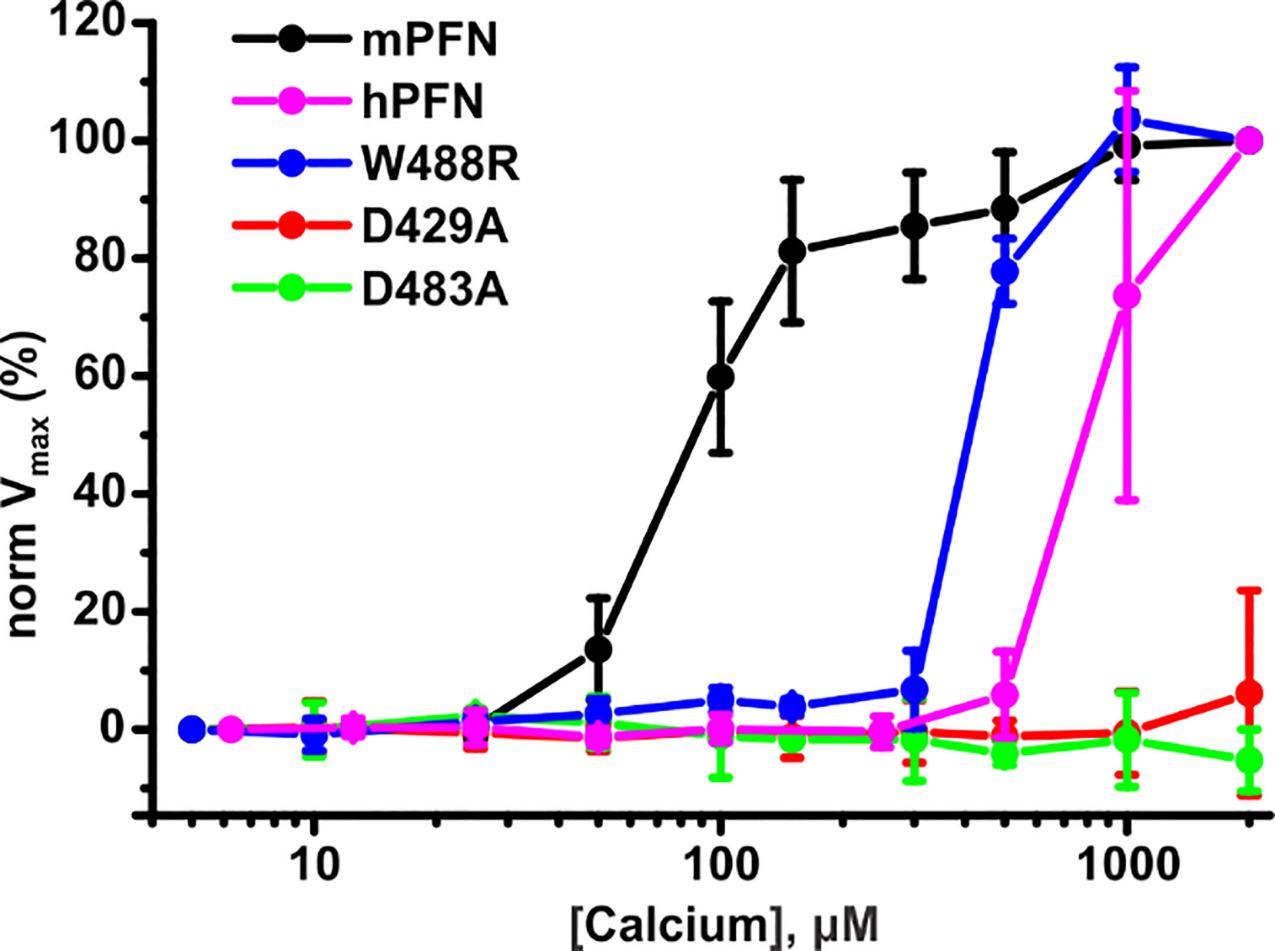
Human perforin (hPFN) lyses erythrocytes at higher [Ca^2+^] compared to mouse perforin (mPFN). Equal concentrations of mPFN and its mutants were admixed with erythrocyte suspension. To this mixture, dilutions of Ca^2+^ were added to test PFN activity. Hemolysis was measured as a drop of turbidity of the erythrocyte suspension as a function of time. Normalized maximal hemolysis (normV_max_) indicates the mean ± SE (n = 3 for mPFN and its mutants and n = 4 for hPFN).

To additionally confirm the SPR and MST results, we investigated the pore-forming activities of hPFN and mPFN in the presence of different [Ca^2+^] as a direct *in situ* measurement of hemolysis velocity, as described before (27). In agreement with previous reports and our findings, EC_50_ for [Ca^2+^] for mPFN is in the low-to-medium micromolar range: 92.3 ± 2.5 μM (n = 3) (**Figure 9**). As shown before (7), the mPFN mutants D429A and D483A do not induce lysis of red blood cells, regardless of [Ca^2+^]. In agreement with the SPR and MST experiments, hPFN exhibited significantly higher EC_50_ for [Ca^2+^] than mPFN: 842.2 ± 40.1 μM (n = 3) (**Figure 9**). Notably, the W488R mutation decreased the affinity of the mPFN C2 domain for Ca^2+^ to a lower affinity, similar to that of hPFN, as its EC_50_ was considerably increased, by ~4.6-fold to 425 ± 8.8 μM (**Figure 9**). Together, these results show that higher [Ca^2+^] is required for hPFN to induce cell lysis in comparison to mPFN.

## Discussion

In this work, we assessed the binding of PFN to lipid membranes by MD simulations and developed biophysical approaches to quantitatively describe the binding of Ca^2+^ to PFN. To evaluate Ca^2+^-binding-induced changes in PFN structure and stability, we designed *in silico* models. Our model (3Ca-mPFN) contained three bound calcium ions observed SmC2P129 (20) and corresponded to three binding sites (out of five) observed in the grove of engineered mouse C2 domain (sites I-III according to terminology in (19)). We omitted a non-canonical binding site observed in the crystal structure of mPFN (10) and engineered mouse C2 domain (19), because it is located at the side of the CBL1 and is not relevant to the membrane interactions of PFN. We also did not include the Ca^2+^ bound to the site IV, which was observed only in the crystal structure of the mouse C2 domain (19). In contrast, the 2Ca-mPFN model included the two calcium ions present in the crystal structure of mPFN (10), which include the non-canonical binding site (site V) and one of the sites in the grove (site II).

The results of the MD simulations showed that the presence of Ca^2+^ reduces the fluctuation of certain amino acids, which reflects an increase in protein stability. These results are in agreement with previous research on the temperature-dependent stability of mPFN, as it is more stable in the presence of Ca^2+^ (20). Similarly, in the case of dysferlin, Ca^2+^ binding greatly increases the temperature-dependent stability of the molecule in solution, which facilitates its interaction with the membrane (24). We observed that the rigidity of the PFN molecule increases with the number of bound Ca^2+^ ions, and the molecule is particularly rigid in the loop areas that surround the Ca^2+^-binding site, i.e., CBL-1 and CBL-3 in the C2 domain. These loops were thus restructured in the model to more closely surround the cation groove with bound ions. This is also characteristic for some other C2 domains (41, 46, 47). In the model with three Ca^2+^ ions, some side chains of amino acid residues were exposed, which were previously identified as important for the interaction of mPFN with the membrane (19, 20).

PFN can interact with the lipid membrane in the absence of Ca^2+^, but with much lower affinity than in the presence of Ca^2+^ (9). Consistently, the mPFN monomer establishes only less stable low-energy interactions in the absence of Ca^2+^ or with two bound Ca^2+^ ions. This confirms that in the crystal structure, only high-affinity Ca^2+^ sites are occupied, which do not play a role in the interaction with the membrane, but probably only in the stabilization of PFN, as is the case with dysferlin (10, 24). In the MD simulation of the monomer with three bound Ca^2+^ ions, we found that the monomer forms stronger interactions with the hydrophobic surface (>10 kcal/mol), especially when it is placed almost perpendicularly (longitudinal axis of PFN) to the membrane surface. Our modelling results revealed that for a stable membrane interaction, at least three Ca^2+^ ions bound to the cation groove between the CBL loops are required. Modelling also showed that, upon its first interaction, the PFN monomer is most likely oriented perpendicular to the membrane tangent, which corresponds to its orientation in the pore (10), as was also proposed previously for mouse C2-domain (19).

To evaluate how mPFN monomers associate with the membrane, we simulated mPFN with three bound Ca^2+^ ions in the most favorable position relative to the membrane in the presence of a bilayer of POPC molecules. We did not observe mPFN insertions into the membrane below the hydrophilic heads of POPC. Shallow membrane insertions were also observed in MD simulations of some other C2-domain-containing proteins, such as cytosolic phospholipase A2, PKCα, and synaptotagmin (48, 49). PFN maintained an upright orientation during the simulation (120 ns in total) (**Figure 3**). The simulation additionally shows that, despite the presence of lipids, PFN formed stable interactions with Ca^2+^ via residues that were also shown to be important for the hemolytic activity of mPFN in *in vitro* experiments (7). Accordingly, the calculated binding enthalpy of hydrophobic residues at the bottom of the molecule (Tyr-486, Trp-488, Trp-427, Thr-431, and Thr-432) decreased significantly.

Molecular modelling therefore shows that Ca^2+^ stabilizes the PFN monomer and changes its conformation to enable protein binding to the lipid membrane. The exposed hydrophobic residues associate with the upper lipid monolayer, and PFN is positioned vertically to the membrane plane. The interactions of PFN with the membrane are strong enough to maintain the association between monomeric PFN and lipids, but do not provide for deeper penetration into the bilayer.

mPFN requires a relatively high [Ca^2+^] (~200 μM) for hemolytic activity (7); however, the exact affinity for Ca^2+^ has not yet been determined for the whole-length protein. The main disadvantage in studying PFN is its poor availability, as standard techniques, such as ITC or NMR, require substantial amounts of protein to determine affinity for ions. Therefore, we used MD and took advantage of biophysical techniques that require low protein consumption, i.e., SPR and MST.

SPR was previously used to assess direct binding of metal ions to proteins (50, 51). SPR can also report conformational changes of ligands on the sensor chip (proteins, aptamers) induced by binding of ions or other small analytes (52–56) or denaturation (57, 58). SPR could, therefore, report the interaction of calcium to PFN directly, if enough Ca^2+^ would bind to change the refractive index of the solution near the sensor chip, or indirectly, through structural changes in the PFN molecule, as concluded from the MD simulations. As PFN binds Ca^2+^ at multiple binding sites, the binding curves obtained by SPR are the combined result of different ion-protein interactions. The affinities obtained from the curves, therefore, represent the total affinity of the Ca^2+^-binding sites of PFN. The steady-state binding curves allow us to describe binding in two [Ca^2+^] regimes (40), the high-affinity region (specific Ca^2+^ binding to the C2 domain) and low-affinity region (binding of Ca^2+^ ions to low-affinity Ca^2+^-binding sites in the C2 domain (19) and other negatively charged groups on the protein or sensor chip). Indeed, the Ca^2+^-titration nuclear magnetic resonance experiment with engineered C2-domain of mouse PFN revealed different affinities for Ca^2+^ for different Ca^2+^-binding sites, with K_D_ values ranging from micromolar (<0.05 mM) to millimolar (5-10 mM) (19). In support of this, the high-affinity binding site was abolished in mPFN mutants D429A (**Figure 5C**) and D483A (**Figure 5D**) and also lacking in the negative controls, BSA and empty flow cell of the sensor chip. To determine the [Ca^2+^] required for PFN to interact with membranes prior to oligomerization, we additionally developed a three-component MST system. In this experiment, we analyzed the change in fluorescence of labelled nanodiscs in presence of PFN along a temperature gradient as a function of [Ca^2+^]. We observed Ca^2+^-dependent changes that were further supported by the appropriate controls, including the data on the mutants D429A and D483A (**Figure 7**). Both approaches gave similar results, albeit SPR reported slightly higher affinities than MST. Lipids are important factors in increasing the affinity of proteins with the C2 domain for Ca^2+^, as they complement the coordination geometry of the ion (45, 59), which is inadequate in PFN. Lipids can act as a plug in the cation groove and prevent Ca^2+^ from escaping into the solution, which could explain the difference in the observed affinities between MST and SPR.

mPFN and hPFN are very similar, as they have 68% identical amino acids. Here, we show that they differ in their affinity for Ca^2+^. Both have an affinity for Ca^2+^ in the micromolar range, which is characteristic of most of the described proteins with C2 domains. mPFN requires lower [Ca^2+^] than hPFN for its cytotoxicity, and this was shown here by both approaches: SPR and MST revealed a ~4-fold and ~6-fold difference, respectively. The two proteins structurally differ in their cation-binding groove. Namely, hPFN contains positively charged arginine (Arg-489) instead of the hydrophobic residue tryptophan (Trp-488 in mPFN). Arginine residues form strong bonds with the phospholipid head groups in some C2 domains (60). Conversely, Trp-488 is important for anchoring PFN to the membrane, according to molecular modelling. Our results show that its substitution with arginine in mPFN reduces the protein’s affinity for Ca^2+^, resulting in binding characteristics that more closely resemble hPFN.

Human killer cells differ from mouse killer cells in their lower cytotoxicity and stability (61), expression of various activating or inhibitory receptors, and location throughout the body (61–63). We have shown that mPFN and hPFN, despite their similar primary structure, differ in their function, hemolytic activity at different [Ca^2+^] (**Figure 9**), and especially affinity for Ca^2+^. In serum and cytosol, [Ca^2+^] is usually ~1.4 mM and <200 nM, respectively (64). The release of PFN from cytotoxic granules into the synaptic cleft would therefore lead to activation of both mPFN and hPFN due to the increase in [Ca^2+^]. Human natural killer cells have therefore developed several mechanisms to protect themselves from such cytotoxic activity, such as precise targeting and tight regulation of cytotoxic protein synthesis (65). Additionally, part of the transformation of the PFN precursor occurs in the endoplasmic reticulum (ER) at physiological pH, where [Ca^2+^] is ~400 μM in unstimulated cells and ~150 μM in stimulated cells (66). Efficient and rapid transport from the ER is thought to prevent PFN from being activated in the ER (5). Unlike human natural killer cells, mouse natural killer cells do not express PFN constitutively but rather when they are stimulated or activated (61). Therefore, human killer cells are more exposed to the action of PFN. Due to decreased negative charge in the area that interacts with Ca^2+^ and thus decreased affinity of hPFN for Ca^2+^, the premature and uncontrolled cytotoxicity of hPFN is additionally limited. We therefore suggest that the lower affinity of hPFN for Ca^2+^ compared to mPFN represents an additional control mechanism of its activity.

## Data availability statement

The raw data supporting the conclusions of this article will be made available by the authors, without undue reservation.

## Author contributions

ON and GA conceived the experiments, ON and GA performed the SPR and MST experiments, ON and GA analyzed the data, FM performed modelling and analyzed the modelling data, GA coordinated experiments, ON and GA wrote the draft of the manuscript. All authors contributed to the article and approved the submitted version.

## In memoriam

This paper is dedicated to the memory of Christopher J. Froelich. Chris was extremely generous in providing the preparations of native human perforin and an ardent supporter of the use of biophysical approaches to study the mechanism of action of perforin.

## References

[1] Voskoboinik I Whisstock JC Trapani JA. Perforin and granzymes: function, dysfunction and human pathology. Nat Rev Immunol (2015) 15(6):388–400. doi: 10.1038/nri3839 25998963

[2] Podack ER Konigsberg PJ. Cytolytic T cell granules. isolation, structural, biochemical, and functional characterization. J Exp Med (1984) 160(3):695–710. doi: 10.1084/jem.160.3.695 6332169 PMC2187410

[3] Podack ER Young JD Cohn ZA. Isolation and biochemical and functional characterization of perforin 1 from cytolytic T-cell granules. Proc Natl Acad Sci USA (1985) 82(24):8629–33. doi: 10.1073/pnas.82.24.8629 PMC390971 2417226

[4] Metkar SS Wang B Aguilar-Santelises M Raja SM Uhlin-Hansen L Podack E. Cytotoxic cell granule-mediated apoptosis: perforin delivers granzyme b-serglycin complexes into target cells without plasma membrane pore formation. Immunity (2002) 16(3):417–28. doi: 10.1016/S1074-7613(02)00286-8 11911826

[5] Lopez JA Jenkins MR Rudd-Schmidt JA Brennan AJ Danne JC Mannering SI. Rapid and unidirectional perforin pore delivery at the cytotoxic immune synapse. J Immunol (2013) 191(5):2328–34. doi: 10.4049/jimmunol.1301205 23885110

[6] Blumenthal R Millard PJ Henkart MP Reynolds CW Henkart PA. Liposomes as targets for granule cytolysin from cytotoxic Large granular lymphocyte tumors. Proc Natl Acad Sci USA (1984) 81(17):5551–5. doi: 10.1073/pnas.81.17.5551 PMC391744 6591203

[7] Voskoboinik I Thia MC Fletcher J Ciccone A Browne K Smyth MJ. Calcium-dependent plasma membrane binding and cell lysis by perforin are mediated through its C2 domain: a critical role for aspartate residues 429, 435, 483, and 485 but not 491. J Biol Chem (2005) 280(9):8426–634. doi: 10.1074/jbc.M413303200 15576364

[8] Lopez JA Susanto O Jenkins MR Lukoyanova N Sutton VR Law RH. Perforin forms transient pores on the target cell plasma membrane to facilitate rapid access of granzymes during killer cell attack. Blood (2013) 121(14):2659–68. doi: 10.1182/blood-2012-07-446146 23377437

[9] Praper T Beseničar MP Istinič H Podlesek Z Metkar SS Froelich CJ. Human perforin permeabilizing activity, but not binding to lipid membranes, is affected by pH. Mol Immunol (2010) 47(15):2492–504. doi: 10.1016/j.molimm.2010.06.001 20580434

[10] Law RH Lukoyanova N Voskoboinik I Caradoc-Davies TT Baran K Dunstone MA. The structural basis for membrane binding and pore formation by lymphocyte perforin. Nature (2010) 468(7322):447–51. doi: 10.1038/nature09518 21037563

[11] Praper T Sonnen A Viero G Kladnik A Froelich CJ Anderluh G. Human perforin employs different avenues to damage membranes. J Biol Chem (2011) 286(4):2946–55. doi: 10.1074/jbc.M110.169417 PMC3024789 20889983

[12] Leung C Hodel AW Brennan AJ Lukoyanova N Tran S House CM. Real-time visualization of perforin nanopore assembly. Nat Nanotechnol (2017) 12(5):467–73. doi: 10.1038/nnano.2016.303 28166206

[13] Ivanova ME Lukoyanova N Malhotra S Topf M Trapani JA Voskoboinik I. The pore conformation of lymphocyte perforin. Sci Adv (2022) 8(6):eabk3147. doi: 10.1126/sciadv.abk3147 35148176 PMC8836823

[14] Naneh O Avčin T Bedina Zavec A. Perforin and human diseases. Subcellular Biochem (2014) 80:221–39. doi: 10.1007/978-94-017-8881-6_11 24798014

[15] Kagi D Ledermann B Burki K Hengartner H Zinkernagel RM. Cd8+ T cell-mediated protection against an intracellular bacterium by perforin-dependent cytotoxicity. Eur J Immunol (1994) 24(12):3068–72. doi: 10.1002/eji.1830241223 7805735

[16] Walsh CM Matloubian M Liu CC Ueda R Kurahara CG Christensen JL. Immune function in mice lacking the perforin gene. Proc Natl Acad Sci USA (1994) 91(23):10854–8. doi: 10.1073/pnas.91.23.10854 PMC45124 7526382

[17] Stepp SE Dufourcq-Lagelouse R Le Deist F Bhawan S Certain S Mathew PA. Perforin gene defects in familial hemophagocytic lymphohistiocytosis. Science (1999) 286(5446):1957–9. doi: 10.1126/science.286.5446.1957 10583959

[18] Vastert SJ van Wijk R D’Urbano LE de Vooght KM de Jager W Ravelli A. Mutations in the perforin gene can be linked to macrophage activation syndrome in patients with systemic onset juvenile idiopathic arthritis. Rheumatology (2010) 49(3):441–9. doi: 10.1093/rheumatology/kep418 20019066

[19] Yagi H Conroy PJ Leung EW Law RH Trapani JA Voskoboinik I. Structural basis for Ca^2+^-mediated interaction of the perforin C2 domain with lipid membranes. J Biol Chem (2015) 290(42):25213–26. doi: 10.1074/jbc.M115.668384 PMC4646173 26306037

[20] Traore DA Brennan AJ Law RH Dogovski C Perugini MA Lukoyanova N. Defining the interaction of perforin with calcium and the phospholipid membrane. Biochem J (2013) 456(3):323–35. doi: 10.1042/BJ20130999 24070258

[21] Cho W Stahelin RV. Membrane binding and subcellular targeting of C2 domains. Biochim Biophys Acta (2006) 1761(8):838–49. doi: 10.1016/j.bbalip.2006.06.014 16945584

[22] Tubiana T Sillitoe I Orengo C Reuter N. Dissecting peripheral protein-membrane interfaces. PloS Comp Biol (2022) 18(12):e1010346. doi: 10.1371/journal.pcbi.1010346 PMC9797079 36516231

[23] Torrecillas A Laynez J Menendez M Corbalan-Garcia S Gomez-Fernandez JC. Calorimetric study of the interaction of the C2 domains of classical protein kinase c isoenzymes with Ca^2+^ and phospholipids. Biochemistry (2004) 43(37):11727–39. doi: 10.1021/bi0489659 15362857

[24] Fuson K Rice A Mahling R Snow A Nayak K Shanbhogue P. Alternate splicing of dysferlin C2a confers Ca(2)(+)-dependent and Ca(2)(+)-independent binding for membrane repair. Structure (2014) 22(1):104–15. doi: 10.1016/j.str.2013.10.001 PMC5993433 24239457

[25] Henkart PA Millard PJ Reynolds CW Henkart MP. Cytolytic activity of purified cytoplasmic granules from cytotoxic rat Large granular lymphocyte tumors. J Exp Med (1984) 160(1):75–93. doi: 10.1084/jem.160.1.75 6736872 PMC2187435

[26] Froelich CJ Turbov J Hanna W. Human perforin: rapid enrichment by immobilized metal affinity chromatography (Imac) for whole cell cytotoxicity assays. Biochem Biophys Res Commun (1996) 229(1):44–9. doi: 10.1006/bbrc.1996.1755 8954081

[27] Naneh O Zavec AB Pahovnik D Zagar E Gilbert RJ Krizaj I. An optimized protocol for expression and purification of murine perforin in insect cells. J Immunol Methods (2015) 426:19–28. doi: 10.1016/j.jim.2015.07.007 26196227

[28] Essmann U Perera L Berkowitz ML Darden T Lee H Pedersen LG. A smooth particle mesh ewald method. J Chem Phys (1995) 103(19):8577–93. doi: 10.1063/1.470117

[29] Jorgensen WL Chandrasekhar J Madura JD Impey RW Klein ML. Comparison of simple potential functions for simulating liquid water. J Chem Phys (1983) 79:926–35. doi: 10.1063/1.445869

[30] Zaccai G. How soft is a protein? a protein dynamics force constant measured by neutron scattering. Science (2000) 288(5471):1604–7. doi: 10.1126/science.288.5471.1604 10834833

[31] Im W Feig M Brooks CL 3rd. An implicit membrane generalized born theory for the study of structure, stability, and interactions of membrane proteins. Biophys J (2003) 85(5):2900–18. doi: 10.1016/S0006-3495(03)74712-2 PMC1303570 14581194

[32] Jo S Kim T Iyer VG Im W. Charmm-gui: a web-based graphical user interface for charmm. J Comp Chem (2008) 29(11):1859–65. doi: 10.1002/jcc.20945 18351591

[33] Klauda JB Venable RM Freites JA O’Connor JW Tobias DJ Mondragon-Ramirez C. Update of the charmm all-atom additive force field for lipids: validation on six lipid types. J Phys Chem B (2010) 114(23):7830–43. doi: 10.1021/jp101759q PMC2922408 20496934

[34] Yang J Yan R Roy A Xu D Poisson J Zhang Y. The I-tasser suite: protein structure and function prediction. Nat Methods (2015) 12(1):7–8. doi: 10.1038/nmeth.3213 PMC4428668 25549265

[35] Baker NA Sept D Joseph S Holst MJ McCammon JA. Electrostatics of nanosystems: application to microtubules and the ribosome. Proc Natl Acad Sci USA (2001) 98(18):10037–41. doi: 10.1073/pnas.181342398 PMC56910 11517324

[36] Humphrey W Dalke A Schulten K. Vmd: visual molecular dynamics. J Mol Graph (1996) 14(1):33–8. doi: 10.1016/0263-7855(96)00018-5 8744570

[37] Schrodinger LLC. The pymol molecular graphics system, version 2.2. (2019).

[38] Shenoy AR Visweswariah SS. Site-directed mutagenesis using a single mutagenic oligonucleotide and dpni digestion of template DNA. Anal Biochem (2003) 319(2):335–6. doi: 10.1016/S0003-2697(03)00286-0 12871732

[39] Bayburt TH Sligar SG. Membrane protein assembly into nanodiscs. FEBS Lett (2010) 584(9):1721–7. doi: 10.1016/j.febslet.2009.10.024 PMC4758813 19836392

[40] Christopeit T Gossas T Danielson UH. Characterization of Ca^2+^ and phosphocholine interactions with c-reactive protein using a surface plasmon resonance biosensor. Anal Biochem (2009) 391(1):39–44. doi: 10.1016/j.ab.2009.04.037 19435596

[41] Corbalan-Garcia S Gómez-Fernández JC. Signaling through C2 domains: more than one lipid target. Biochim Biophys Acta (2014) 1838(6):1536–47. doi: 10.1016/j.bbamem.2014.01.008 24440424

[42] Murray D Honig B. Electrostatic control of the membrane targeting of C2 domains. Mol Cell (2002) 9(1):145–54. doi: 10.1016/S1097-2765(01)00426-9 11804593

[43] Ritchie TK Grinkova YV Bayburt TH Denisov IG Zolnerciks JK Atkins WM. Chapter 11 - reconstitution of membrane proteins in phospholipid bilayer nanodiscs. Meth Enzymol (2009) 464:211–31. doi: 10.1016/S0076-6879(09)64011-8 PMC4196316 19903557

[44] Li L Shin OH Rhee JS Araç D Rah JC Rizo J. Phosphatidylinositol phosphates as Co-activators of Ca^2+^ binding to C2 domains of synaptotagmin 1. J Biol Chem (2006) 281(23):15845–52. doi: 10.1074/jbc.M600888200 16595652

[45] Radhakrishnan A Stein A Jahn R Fasshauer D. The Ca^2+^ affinity of synaptotagmin 1 is markedly increased by a specific interaction of its C2b domain with phosphatidylinositol 4,5-bisphosphate. J Biol Chem (2009) 284(38):25749–60. doi: 10.1074/jbc.M109.042499 PMC2757977 19632983

[46] Wang Y Tadayon R Santamaria L Mercier P Forristal CJ Shaw GS. Calcium binds and rigidifies the dysferlin C2a domain in a tightly coupled manner. Biochem J (2021) 478(1):197–215. doi: 10.1042/BCJ20200773 33449082

[47] Morales KA Yang Y Cole TR Igumenova TI. Dynamic response of the C2 domain of protein kinase cα to Ca(2+) binding. Biophys J (2016) 111(8):1655–67. doi: 10.1016/j.bpj.2016.09.008 PMC5071625 27760353

[48] Lai CL Landgraf KE Voth GA Falke JJ. Membrane docking geometry and target lipid stoichiometry of membrane-bound pkcalpha C2 domain: a combined molecular dynamics and experimental study. J Mol Biol (2010) 402(2):301–10. doi: 10.1016/j.jmb.2010.07.037 PMC3602446 20659476

[49] Ward KE Bhardwaj N Vora M Chalfant CE Lu H Stahelin RV. The molecular basis of ceramide-1-Phosphate recognition by C2 domains. J Lipid Res (2013) 54(3):636–48. doi: 10.1194/jlr.M031088 PMC3617939 23277511

[50] Wu C-M Lin L-Y. Immobilization of metallothionein as a sensitive biosensor chip for the detection of metal ions by surface plasmon resonance. Biosens Bioelectronics (2004) 20(4):864–71. doi: 10.1016/j.bios.2004.03.026 15522603

[51] Wu C-M Lin L-Y. Utilization of albumin-based sensor chips for the detection of metal content and characterization of metal–protein interaction by surface plasmon resonance. Sensors Actuators B (2005) 110(2):231–8. doi: 10.1016/j.snb.2005.01.047

[52] Dell’Orco D Koch K-W. Fingerprints of calcium-binding protein conformational dynamics monitored by surface plasmon resonance. ACS Chem Biol (2016) 11(9):2390–7. doi: 10.1021/acschembio.6b00470 27380526

[53] Dejeu J Bonnet H Spinelli N Defrancq E Coche-Guérente L van der Heyden A. Impact of conformational transitions on spr signals–theoretical treatment and application in small Analytes/Aptamer recognition. J Phys Chem C (2018) 122(37):21521–30. doi: 10.1021/acs.jpcc.8b07298

[54] Gestwicki JE Hsieh HV Pitner JB. Using receptor conformational change to detect low molecular weight analytes by surface plasmon resonance. Anal Chem (2001) 73(23):5732–7. doi: 10.1021/ac0105888 11774914

[55] Geitmann M Danielson UH. Studies of substrate-induced conformational changes in human cytomegalovirus protease using optical biosensor technology. Anal Biochem (2004) 332(2):203–14. doi: 10.1016/j.ab.2004.06.008 15325287

[56] Flatmark T Stokka AJ Berge SV. Use of surface plasmon resonance for real-time measurements of the global conformational transition in human phenylalanine hydroxylase in response to substrate binding and catalytic activation. Anal Biochem (2001) 294(2):95–101. doi: 10.1006/abio.2001.5163 11444803

[57] Zako T Harada K Mannen T Yamaguchi S Kitayama A Ueda H. Monitoring of the refolding process for immobilized firefly luciferase with a biosensor based on surface plasmon resonance. J Biochem (2001) 129(1):1–4. doi: 10.1093/oxfordjournals.jbchem.a002818 11134950

[58] Sota H Hasegawa Y Iwakura M. Detection of conformational changes in an immobilized protein using surface plasmon resonance. Anal Chem (1998) 70(10):2019–24. doi: 10.1021/ac9713666 9608841

[59] Hirano Y Gao Y-G Stephenson DJ Vu NT Malinina L Simanshu DK. Structural basis of phosphatidylcholine recognition by the C2–domain of cytosolic phospholipase A2α. eLife (2019) 8:e44760. doi: 10.7554/eLife.44760 31050338 PMC6550875

[60] Ochoa WF Garcia-Garcia J Fita I Corbalan-Garcia S Verdaguer N Gomez-Fernandez JC. Structure of the C2 domain from novel protein kinase cepsilon. a membrane binding model for Ca(2+)-independent C2 domains. J Mol Biol (2001) 311(4):837–49. doi: 10.1006/jmbi.2001.4910 11518534

[61] Sungur CM Murphy WJ. Utilization of mouse models to decipher natural killer cell biology and potential clinical applications. Hematology (2013) 2013:227–33. doi: 10.1182/asheducation-2013.1.227 24319185

[62] Crome SQ Lang PA Lang KS Ohashi PS. Natural killer cells regulate diverse T cell responses. Trends Immunol (2013) 34(7):342–9. doi: 10.1016/j.it.2013.03.002 23601842

[63] Abel AM Yang C Thakar MS Malarkannan S. Natural killer cells: development, maturation, and clinical utilization. Front Immunol (2018) 9:1869. doi: 10.3389/fimmu.2018.01869 30150991 PMC6099181

[64] Dudev T Lim C. Competition among metal ions for protein binding sites: determinants of metal ion selectivity in proteins. Chem Rev (2014) 114(1):538–56. doi: 10.1021/cr4004665 24040963

[65] Prager I Watzl C. Mechanisms of natural killer cell-mediated cellular cytotoxicity. J Leukoc Biol (2019) 105(6):1319–29. doi: 10.1002/JLB.MR0718-269R 31107565

[66] Arnaudeau S Kelley WL Walsh JV Jr. Demaurex N. Mitochondria recycle Ca(2+) to the endoplasmic reticulum and prevent the depletion of neighboring endoplasmic reticulum regions. J Biol Chem (2001) 276(31):29430–9. doi: 10.1074/jbc.M103274200 11358971

